# Whole exome sequencing identifies a novel homozygous frameshift mutation in the ASPM gene, which causes microcephaly 5, primary, autosomal recessive

**DOI:** 10.12688/f1000research.12102.1

**Published:** 2017-12-21

**Authors:** Desaraju Suresh Bhargav, N. Sreedevi, N. Swapna, Soumya Vivek, Srinivas Kovvali

**Affiliations:** 1 Unit for Human Genetics, All India Institute of Speech and Hearing, Manasagangothri, India; 2 Department of Clinical Services, All India Institute of Speech and Hearing, Manasagangothri, India; 3 Department of Speech Language Pathology, All India Institute of Speech and Hearing, Manasagangothri, India

**Keywords:** Microcephaly, ASPM, Exome Sequencing, MCPH5, MCPH

## Abstract

Microcephaly is a genetically heterogeneous disorder and is one of the frequently notable conditions in paediatric neuropathology which exists either as a single entity or in association with other co-morbidities. More than a single gene is implicated in true microcephaly and the list is growing with the recent advancements in sequencing technologies. Using massive parallel sequencing, we identified a novel frame shift insertion in the abnormal spindle-like microcephaly-associated protein gene in a client with true autosomal recessive primary microcephaly.  Exome sequencing in the present case helped in identifying the true cause behind the disease, which helps in the premarital counselling for the sibling to avoid future recurrence of the disorder in the family.

## Introduction

Microcephaly with no other anomalies in the brain structure is termed as true microcephaly or autosomal recessive primary microcephaly (MCPH), where the pathology of brain is generally congenital and static with mild to moderate intellectual disability (ID) ( http://www.orpha.net/consor/cgi-bin/OC_Exp.php?Expert=2512). Microcephaly is seen in numerous syndromes ^1^ and even in true microcephaly, there is possibility of more than one gene implicated ^2, 3^, thus screening for a single gene may not be very fruitful in this population. Recently, whole exome sequencing (WES) has emerged as a potential approach to delineate the molecular pathology in the microcephaly population with ID ^1^. Using WES, we report a *de novo* frame shift (insertion) mutation in the calponin-homology domain of *ASPM* (Abnormal Spindle-Like, Microcephaly-Associated) gene which is a candidate for MCPH5 (Microcephaly 5, primary, autosomal recessive) in a client with true microcephaly.

## Report

**Figure 1.**
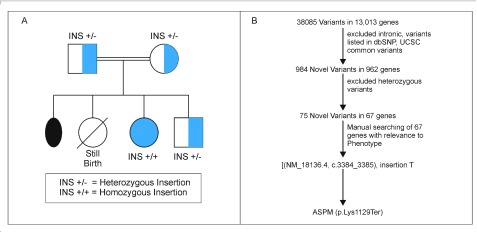
( **A**) The pedigree of the family; ( **B**) workflow of the variant prioritization.

The client sequenced was under the research project cerebral palsy and spectrum conditions (seizures, mental retardation, microcephaly and other neurodevelopmental disorders), which aims to find disease causing mutations in a sample of 100 clients recruited from our Motor Speech Disorders Clinic at Department of Clinical Services, All India Institute of Speech and Hearing. This is a research project and hence ethical clearance was obtained, reference was mentioned in *Methods*. The client is a 15 year old female born out of a consanguineous union (parents were first cousins) diagnosed with microcephaly (occipitofrontal head circumference was 40 centimetres) and developmental delay. The mother had a history of one miscarriage at the third month of gestation and a still birth (female) at the eighth month ( Figure 1A). An ultra sound scan of the foetus (client) at the eight month of pregnancy revealed delayed development. The client was born at term through normal delivery with no birth trauma. Her younger brother was clinically normal. The client had a squint at birth and had mild ID. At age 12 years, her developmental age was between 66 to 78 months as assessed by the Developmental Screening Test (DST) ^4^. Her Receptive Language Age (RLA) and Expressive Language Age (ELA) was 18–20 months as revealed by Receptive Expressive Emergent Language Scale (REELS) ^5^. She had a vocabulary of around 50 words and was able to comprehend commands, would recognize family members and common objects. She expressed herself through single word utterances, gestures and pointing.

### Sequencing methodology

The study was approved by the Institutional Ethical Body, All India Institute of Speech and Hearing [Ethical clearance reference number: SH/CDN/ARF-40/2016-17]. After obtaining written informed consent from the parents of the client, 5 ml of blood was collected from all family members (mother, father and brother) into EDTA coated vacutainers and DNA was isolated using Pure Link Genomic DNA Isolation Kit (Thermo Fisher Scientific), as per the manufacturer’s instructions. Approximately 100ng of genomic DNA was used to construct Exome libraries using Ion Ampliseq Exome RDY Panel (Thermo Fisher Scientific), as per the manufacturer’s protocol and these were quantified using High Sensitivity genomic DNA Assay on Qubit 3.0 (Thermo Fisher Scientific). Approximately 25 Pico moles of the library was used with the Ion Chef Instrument (Thermo Fisher Scientific) for template generation followed by, enrichment of templated ion sphere particles. Sequencing was performed using Hi-Q chemistry on Ion Proton system (Thermo Fisher Scientific) at our facility. Two samples were sequenced per chip and run generated 10.98 GB. The sample in question yielded 28,105,510 reads with 185 mean base pair length.

## Results

### For the client sample

QC filtered reads were aligned to reference genome GRCh37/hg19. Of the 28.1 million reads, 99.27% reads were on target with a mean genomic coverage of 91.83%. Mean depth was 81.32x with 89.87% mean uniformity. Variants were called from raw data using inbuilt variant caller plug-in present in Torrent suite (Version 5.2.2). Ion Reporter (version 5.4) annotated 38,085 variants from 13,013 genes to hg19 from the VCF file generated by variant caller plug-in. Variant prioritization was performed as shown in Figure 1B.

From the pedigree chart, we assumed autosomal recessive inheritance and filtered out heterozygous variants. This resulted in 75 homozygous variants from 67 genes. We found a novel frame shift insertion in exon 13 of the *ASPM* gene [(NM_018136.4), c.3384_3385 Insertion T], which induces a termination codon (p.Lys1129Ter) leading to non functional ASPM protein. Insertion was verified by Sanger sequencing using BDTv3 on 3500 Genetic Analyzer (Thermo Fisher Scientific). Homozygous insertion was confirmed in the client. Both parents and unaffected sibling were found to be heterozygous carriers ( Figure 1A).

## Discussion

ASPM protein determines cerebral cortical size. During initial stages of corticogenesis, the ASPM protein is essential in facilitating the proliferation of neural progenitors ^6^. This process determines the cerebral cortical volume ^7^ which has tripled over the last ~ 2 million years, leading to exceptionally big brain in humans compared to their primate counterparts ^8^. This increase in the human brain size is believed to be one contributing factor for the emergence of higher cognitive function and language ability that are restricted to humans ^8^. So far, 17 genes have been reported in which, mutations lead to the development of MCPH ^3, 4, 9– 26^. The phenotype(s) arising from pathogenic variants in these 17 genes are each named from MCPH 1 – MCPH 17 (there are 17 genes identified so far that cause autosomal recessive primary microcephaly, MCPH arising from these 17 genes are termed from MCPH 1 to MCPH 17) and the majority of the genetic load in MCPH is contributed by the *ASPM* gene, making MPCH5 the most prevalent of all the types of MCPH. Frame shift and protein truncating mutations in *ASPM* cause MCPH5 and these mutations are restricted to be seen in homozygous state only in the MCPH5 population ^27, 28^ (i.e., heterozygous mutations does not have any effect and only homozygous mutations will cause the MCPH).

## Conclusion

The novel insertion mutation found in the *ASPM* gene in the present study segregated with the phenotype in the family, establishes the role of the novel frame shift mutation identifiedin the development of MCPH5 in the case studied. Candidate gene study by Sanger sequencing is time consuming and not economical when compared to WES. Given its higher diagnostic yield as evident by published studies on neurodevelopmental disorders ^2^ and also from the present work, we support the findings reported by *Rump et al.* (2016) which states that WES in microcephaly population will end unnecessary further evaluations and aid in early appropriate interventions ^2^.

## Consent

Written informed consent to carry out the study and for the publication of the client’s and client’s sibling’s clinical details were obtained from the parents. Clinical details were obtained from the parents of the client.

## Data availability

Sequence data for the insertion mutation (client sample) was deposited in Genbank under accession number MG063723.
