# Supplementary material for: Whole exome sequencing identifies a novel homozygous frameshift mutation in the ASPM gene, which causes microcephaly 5, primary, autosomal recessive
Source: F1000Res. 2017 Dec 21;6:2163. [Version 1] doi: 10.12688/f1000research.12102.1 (PMC5770997; doi:10.12688/f1000research.12102.1)
Supplement: Peer review history

# Peer review: Referee response for version 1

**DOI:** 10.5256/f1000research.13096.r29314

**Published:** 2018-01-15

**Authors:** Sulman Basit

**Affiliations:** 1 Center for Genetics and Inherited Diseases, Taibah University, Almadinah Almunawwarah, Saudi Arabia

The manuscript by Bhargav and colleagues describes a novel homozygous frameshift insertion mutation in a known microcephaly gene in a family segregating autosomal recessive primary microcephaly. 

 Whole exome sequencing was used to identify a mutation underlying MCPH phenotype. 

 Overall, the manuscript is written well. Authors have presented the data in a professional way and I have

 no reservation to approve this submission.

I have read this submission. I believe that I have an appropriate level of expertise to confirm that it is of an acceptable scientific standard.

## References

[1] MemonMMRazaSIBasitSKousarRAhmadWAnsarM: A novel WDR62 mutation causes primary microcephaly in a Pakistani family. *Mol Biol Rep*.2013;40(1) : 10.1007/s11033-012-2097-7 591-5 10.1007/s11033-012-2097-7 23065275

[2] KousarRHassanMJKhanBBasitSMahmoodSMirAAhmadWAnsarM: Mutations in WDR62 gene in Pakistani families with autosomal recessive primary microcephaly. *BMC Neurol*.2011;11: 10.1186/1471-2377-11-119 119 10.1186/1471-2377-11-119 21961505PMC3196702

[3] HashmiJAAl-HarbiKMRamzanKAlbalawiAMMehmoodASammanMIBasitS: A novel splice-site mutation in the ASPM gene underlies autosomal recessive primary microcephaly. *Ann Saudi Med*.36(6) : 10.5144/0256-4947.2016.391 391-396 10.5144/0256-4947.2016.391 27920410PMC6074201

[4] BasitSAl-HarbiKMAlhijjiSAAlbalawiAMAlharbyEEldardearASammanMI: CIT, a gene involved in neurogenic cytokinesis, is mutated in human primary microcephaly. *Hum Genet*.2016;135(10) : 10.1007/s00439-016-1724-0 1199-207 10.1007/s00439-016-1724-0 27519304

# Peer review: Referee response for version 1

**DOI:** 10.5256/f1000research.13096.r29354

**Published:** 2018-01-12

**Authors:** Shahid Mahmood Baig

**Affiliations:** 1 National Institute for Biotechnology and Genetic Engineering, Pakistan Institute of Engineering & Applied Sciences, Faisalabad, Pakistan

In this report, a novel frameshift insertion in exon 13 of the ASPM gene in an Indian patient with autosomal recessive primary microcephaly has been reported. 

 This finding adds to the spectrum of mutations in ASPM gene using whole exome sequencing (WES) for premarital screening and prenatal diagnosis to prevent affected births in at risk pregnancies. This report justifies the use of WES in delineating MCPH as compared to candidate gene approach. It is a descent short report, and hence recommended for indexing in its present form.

I have read this submission. I believe that I have an appropriate level of expertise to confirm that it is of an acceptable scientific standard.
